# Chicago Society of Physicians and Surgeons

**Published:** 1875-04

**Authors:** Ralph E. Starkweather


### Reports of Societies.

### CHICAGO SOCIETY OF PHYSICIANS AND SURGEONS.

*Regular Meeting, March* 22, 1875.

(Reported by RALPH E. STARKWEATHER, M.D.)

The Society met as usual, at the Grand Pacific Hotel, the President, Dr. John Bartlett, in the chair.

Dr. Etheridge, of the Section on Materia Medica and Therapeutics, presented a written report, of which the following is a summary:

### SULPHIDE OF CARBON IN THE TREATMENT OF ATONIC ULCERS AND CHRONIC ULCERATION.

After adverting to the proverbial difficulty of successful treatment of ulcers, mention was made of their common features, namely, atony and chronicity, with a tendency to extension and a total absence of reparative nature and cicatrization. The sulphide of carbon was used in ulcerations first in 1867, by M. Michel, while he was an interne in Saint Lazare. Its nauseous odor and its toxic action are the predominant characteristics, and have doubtless kept it from being extensively used.

By the German physicians, this agent has been employed in the treatment of neuralgias and arthralgias, probably because of its refrigerant and anæsthetic action. Compresses soaked in carbic sulphide in some aromatic essence were placed over the painful part, and renewed from time to time till relief followed. Its vapors mixed with those of iodine have been used in certain affections of the eye and ear. It has been used also upon the erysipelatous redness of chilblains.

Dr. Guillaumet also gives details of three cases of ulcers selected from a large number thus treated.

Case 1. Large ulceration of the right labium majorum of four and a half months duration, in a syphilitic subject. Cure in fifteen days.

Alice V., aged 23. Upon the inner aspect of the right labium there is a large ulceration, extending nearly its whole length, measuring about one and a quarter inches in breadth. It is atonic, has a grayish base, irregular serrated borders, and a depth of about one-fifth of an inch. The various treatment adopted by others was of no avail, the ulceration continued to spread. She came under observation four and a half months after the ulcer appeared. The sulphide of carbon was at once employed with gratifying success. Thirteen applications in fifteen days sufficed to produce complete cicatrization of the ulcer. After the first application of this agent there is no appreciable change in the aspect of the ulcer. Upon the fifth or sixth application, a sensible amelioration and a very rapid course of cicatrization followed. It is necessary to be careful in using the sulphide, because of its extreme volatility and nauseous odor. It may be applied as follows: The bottle containing pure sulphide of carbon is held very near to the ulcer to be dressed; a piece of charpie is soaked in the liquid and squeezed upon the mouth of the bottle to expel any excess of the drug; then the piece of charpie is lightly and rapidly brushed over the surface of the ulcer which is immediately covered with a powder of bismuth subnitrate or of starch. The pain of the application is short, quick and very fugacious. The pain diminishes after a few applications have been made. As a rule, its intensity may be said to be in inverse ratio to the number of the dressings. The amount of the sulphide to be used should be proportional to the duration and vitality of the ulcer. If too much be used, cicatrization might be retarded.

*Resume:*

I. Sulphide of carbon is a powerful cicatrizer.

II. Its action is limited and rapid; it is wholly local, and does not produce a single accident which follows the inhalation of its vapors.

III. Its application is accompanied with pain, sometimes very severe—proportional to the susceptibility of the patient—but is of very short duration in most patients, followed immediately by a period of anæsthesia which is not constant.

IV. Sulphide of carbon acts upon ulcers of various kinds (syphilitic, scrofulous, diphtheritic, etc.,) and changes them all beneficially.

V. This is a valuable agent in the treatment of wounds or ulcers, presenting the common characteristics of atony and chronicity.

### ERGOTINE.

Dr. E. R. Squibb speaks as follows of ergotine:

The existence of one or more active principles, which may be isolated for therapeutic use, has been very generally believed, hence various substances have been extracted and sold under the name of ergotine by different makers, the dose and properties being equally diverse. Dr. Squibb, however, explains that he does not believe that there is an active principle separable from ergot, which in any proper sense represents the drug, and that there is no such thing as ergotine. Of course certain of its constituents, such as lignin, starch, fixed oil, gum, etc., are known to be inert, and may be excluded by choice of a proper menstruum.

THE AQUEOUS AND ALCOHOLIC SOLUTIONS OF ERGOT.

These differ in their effects on the circulatory appa ratus. The aqueous solution excites the activity of the cardiac inhibitory centres and the vaso-motor centre in the medulla ; it slackens the pulse, narrows the calibre of the small arteries, and increases the blood pressure. Very large doses paralyze the heart at once, and so effectually that the induced current fails to restore it. Such effects are not at all produced by the alcoholic solution. The latter produces symptoms akin to those of acro-narcotic poisoning, viz., irritation of the mucous membrane of the stomach and bowel, tonic cramps, agitation.

THE TREATMENT OF VARICOSE VEINS BY ERGOTINE.

Dr. Etheridge quoted from the Berlin Weekly Clinic several interesting cases of the above in which the treatment had been successful. The most generally accepted idea, is, as to the modus medendi of ergot subcutaneously, that diminution of the calibre of vessels is produced by the drug stimulating the smooth muscle of the veins and arteries to contract. In its use in varicose veins it doubtless partially produces diminution of the capacity of the veins by producing inflammatory infiltration above mentioned, which directly compresses the veins.

Dr. Max Schuler states as a result of many experiments that ergot produces contraction of arterioles. The smaller the vessel the larger and firmer is the contraction. Arteries increase proportionally in smooth muscular fibre as they become smaller. From this fact it is inferred that ergot exerts its effect on this muscular fibre, not necessarily through the vaso-motor nerves, for the same effect, viz., muscular contraction, could be secured in a part injected with a solution of ergot, after the sympathetic nerve supplying the part had been cut.

Ergot by the stomach will produce none of the effects that can be called hæmostatic; subcutaneously it will produce them.

Dr. Bevan, of the Section on Clinical Reports, presented in writing several cases of interest, supplementing one of these by instructive details of the post mortem examination of the patient. The cases were limited to those of the nervous system.

The first case was that of a patient who was a Danish laborer, fifty years of age, with chronic myelitis. A year before admission to the County Hospital, while at work, he slipped from a ladder, falling sixteen feet, and struck upon his back. From time to time he had been under treatment, but as often the pain in back and side had returned. Walking became impossible, and he grew weaker. On examination, no tenderness along the spine; has to be supported when he walks—and holds the lower portion of the spine in a fixed position—gait is rotary. The treatment was seven grains of the potassium iodide with $\frac{1}{30}$ gr. strych. sulphate in bitter infusion three times a day—after twenty days, treatment was changed, and one-half fluid drachm of ext. ergotæ fluid was exhibited three times a day—a lotion of equal parts tr. iodine and glycerine was applied to the spine and sacrum. The improvement was speedy and marked—motion of legs pretty fair—pain is slight, sleep and appetite greatly improved.

Dr. Bevan next gave the history and treatment of a case of subacute meningitis. Dr. Bevan then reported a case of epilepsia. The fourth case cited was also one of epilepsia and in a patient thirty-five years of age, an Englishwoman—of which the particulars are briefly as follows: For upwards of seventeen years the patient has had epileptic seizures, the intervals sometimes being so long as eight months. She has borne several healthy children—the catamenial functions have been normal.

An epileptic attack would come on somewhat thus: while sitting near the fire, she would drop into a partially insensible condition, resembling sleep, from which it would be impossible to arouse her. This stupor would continue ten to twelve hours, and be so profound that she has been moved in a carriage several miles unknown to herself. Pain and weight in the top of the head have lately troubled her—emaciation progresses. She never asks for food; but if awake, will take it when it is presented to her. Pulse would be 72, respiration 12, during one of these seizures; muscles flaccid, occasionally twitching; jaws firmly closed. The treatment which gave her prompt relief was the potassium bromide.

Shortly before discharge from the hospital a tonic was ordered: ℞. Strychniæ Sulphatis, gr. $\frac{1}{60}$; Acidi Nitrici Diluti, gtt. v; Infus. Gentian, f. ℥ ss. M. Take the same three times a day.

During the discussion of the above papers, Dr. Bevan mentioned a case of diabetes mellitus which he had treated with excellent results by the use of the fluid ext. of ergot in doses of one fluid drachm four times a day. The amount of urine voided was sixteen pints daily; ten weeks after beginning treatment the amount of urine daily voided was only four pints, and very much less saccharine; the thirst and emaciation of the patient had become much less.

Dr. Owens queried whether there was any disease ergot would not relieve, and quoted a case of diarrhœa of several months duration which he relieved by using the wine of ergot. In regard to the sulphide of carbon, his experience in its use upon ulcers had been satisfactory. One patient had an ulcer upon each leg—he treated one of the ulcers by elevating the limb and warm water dressings; the other, he treated with the sulphide of carbon, and healed the ulcer quickly. The time lost by the first method of treatment was over two weeks.

Dr. Hyde thought that in many instances chronic ulcers could be treated in no way more efficaciously or expeditiously than by cross strapping and starch bandage.

Dr. Powell mentioned two methods of treating ulcers of the leg—one by transplantation of skin; the other by half-inch incisions at intervals around the entire ulcer. It would not be sufficient merely to remove epithelial scales—actual integument was needed—a piece the one-sixteenth of a square inch would suffice to make a dozen insertions—each insertion would form a centre for granulation, which would cover about an inch. Upon the coalescence of these various islands of healthy skin, the ulcer would be healed. Little cuts should be made in the floor of the ulcer, and a piece of sound skin inserted.

Dr. Hollister adverted to a method formerly used by Dr. Brainard, that of placing upon the ulcer a piece of lint sprinkled with a little iodine.

Dr. Hyde had had good results in cases of ulcers from indolent buboes in the employment, after the above method, of bromine.

Dr. P. S. Hayes said that in one of the preparations of ergot, acetic acid was used, about one per cent. being added to the officinal fluid extract, which rendered the liquid preparation more permanent; the acid has the effect of removing all inert matter; ergotine is not a simple body like the morphia sulphate.

Dr. Powell, in a case of double popliteal aneurism, had employed hypodermic injections of ergotine upwards of five weeks, with no good results; one of the tumors even enlarged.

Dr. Etheridge remarked, that in treating varicose veins, the injections of ergotine would be useless unless made near the varix. The local inflammation produced by the irritating alcohol might be sufficient to diminish the tumor.

He never recommended the alcoholic solution for hypodermic injections; the alcoholic and aqueous solutions have no one effect in common.

One grain of the aqueous extract represents one minim—this properly diluted may be used for hypodermic injections.

The discussion was continued by Doctors Merriman, Davis and Henrotin, with reports of cases.

